# Effectiveness of nanosilver fluoride and silver diamine fluoride in arresting early childhood caries: a randomized controlled clinical trial

**DOI:** 10.1186/s12903-024-04406-3

**Published:** 2024-06-18

**Authors:** Maryam Quritum, Ahmed Abdella, Hala Amer, Lubna M. El Desouky, Maha El Tantawi

**Affiliations:** 1 Department of Pediatric Dentistry and Dental Public Health, Faculty of Dentistry, Alexandria University, Champolion St, 21527 Azarita, Alexandria, Egypt; 2 Department of Pharmaceutics, Faculty of Pharmacy, Alexandria University, Alexandria, Egypt

**Keywords:** Caries arrest, Early childhood caries, Nano silver fluoride, Silver diamine fluoride, Children, Primary teeth

## Abstract

**Background:**

One of the most prevalent health problems affecting children worldwide is untreated caries in primary teeth. Agents to arrest caries are used to manage untreated decay in children in disadvantaged communities. Nano Silver Fluoride (NSF) overcomes the staining problems of Silver Diamine Fluoride (SDF). This study compared the clinical cariostatic effect of NSF to 38% SDF for arresting caries lesions.

**Methods:**

The study included 360 children younger than 4 years, with at least one active lesion, ICDAS score ≥ 3, recruited from nurseries in a rural area in Alexandria, Egypt, in 2022. They were randomly assigned to receive a single application of NSF at baseline, or two applications of SDF at baseline and after 6 months. The arrest of active carious lesions was assessed after 6 and 12 months using ICDAS criteria, and parents’ satisfaction with child appearance was also assessed. Chi-Square test was used to compare the groups and multi-level multiple logistic regression was used to assess the effect of the intervention on caries arrest at lesion level and binary logistic regression was used to assess the effect at patient level.

**Results:**

1853 active lesions were included in children whose mean (SD) age was 42.3 (8.2) months. The arrest rate was significantly higher in the NSF than the SDF group at lesion level (78.4% and 65.0% at 6 months and 71.3% and 56.3% at 12 months, *p* < 0.001). In regression analysis, NSF had significantly higher odds of caries arrest than SDF at lesion level (at 6 months, AOR = 2.57, 95% CI: 1.55, 4.26 and at 12 month, AOR = 3.27, 95% CI: 1.89, 5.67). Parents of children receiving NSF had significantly greater satisfaction with their children’s dental appearance than those receiving SDF: (97.2% and 76.1%, respectively, *p* < 0.001).

**Conclusion:**

NSF demonstrated greater effectiveness in arresting caries in preschool children without inducing black staining of teeth and with greater parental satisfaction than SDF. NSF can be an alternative to SDF in arresting caries especially in underprivileged communities.

**Trial registration:**

The trial was registered in the clinicaltrials.gov registry (#NCT05255913)-16/02/2022.

## Background

Early childhood caries (ECC), defined as any decayed or filled or missing due to caries tooth in children under 6-years of age, is a major public health problem [1]. According to the Global Burden of Disease study, untreated decay in primary teeth was the 10th most prevalent condition globally [2], with 532 million children affected worldwide in 2017 [3]. ECC does not only affect teeth, but the consequences of this disease have negative impact on growth, wellbeing, and quality of life of affected children [4]. Moreover, ECC has a considerable economic burden caused by the need for oral rehabilitation under general anesthesia [5] which poses high risks for children, burden for families, and substantial expenses on healthcare systems [6].

Minimally invasive methods for preventing and arresting caries present affordable options to manage caries in deprived communities, where dental workforce shortage and limited availability of sophisticated dental equipment are concerns [7, 8]. Various fluoride agents have excellent cariostatic efficacy [9] including silver diamine fluoride (SDF) which combines sodium fluoride’s ability to remineralize tooth structure with silver nitrate’s antimicrobial properties against cariogenic bacteria. SDF can be used outside the clinic setting without dental instruments or caries removal [10, 11] with proven efficacy in arresting ECC [12]. However, its major drawbacks are black staining of carious lesions and a metallic taste [13]. In addition, accidental contact with SDF solution causes reversible, mildly painful lesions in the oral mucosa that usually resolve within 48 h [14].

Nanotechnology is a new method to manage caries and remineralize dental tissues [15]. Silver nanoparticles (AgNPs) have potent antimicrobial properties against a wide variety of microorganisms [16–18]. Nano Silver Fluoride (NSF) contains AgNPs, chitosan, and fluoride with preventive and antimicrobial properties and no black staining [19]. NSF is safe, eco-friendly and of low cost [20, 21]. AgNPs’ antibacterial action is based on their ability to penetrate the bacterial cell wall due to the particles’ great surface area with greater contact with microorganisms. This damages the cell membrane and interferes with DNA replication leading to bacterial cell death [22]. Evidence showed the anti-caries property and better efficacy of NSF than the control in ECC arrest [19, 23, 24].

In Egypt, ECC prevalence ranges from 61.4% [25] to 69.2% [26]. This high prevalence threatens the welfare of children with impact on the economy and sustainable development. There is limited research comparing the effect of NSF and SDF in arresting caries clinically. This study compared the effectiveness of NSF and 38% SDF in arresting ECC in preschool children in a rural area in Egypt; a setting with high disease burden and great need for dental care. The null hypothesis was that there would be no significant difference between the two agents in arresting ECC.

## Methods

This study was a randomized controlled clinical trial with two parallel arms conducted in nurseries in rural areas near Alexandria, Egypt in 2022. Ethical approval was obtained from the Research Ethics Committee, Faculty of Dentistry, Alexandria University, Egypt (0265-07/2021). The study was conducted following the Helsinki declaration guidelines for human research [27]. Parents gave written informed consent before the study after detailed explanation of the objectives, benefits and risks. They also received instructions about oral hygiene and proper diet. The trial was recorded in the clinicaltrials.gov registry (#NCT05255913) and reported following the CONSORT guidelines [28]. Participants were referred for treatment if their teeth showed caries progression after the intervention.

### Participants

Children were included if they were ≤ 4 years old with at least one active carious lesion in a primary tooth, with score 3 or higher according to the International Caries Detection and Assessment System- ICDAS detected using visual and tactile inspection to assess lesion severity and activity [29, 30]. Exclusion criteria were teeth that caused spontaneous pain or showed signs of pulpal infection, swelling and/ or abscess. We excluded children with a history of major systemic disorders or allergy to silver or any of the materials used in the study and children with intellectual disabilities. All eligible teeth per child were included.

### Intervention

The children received either 38% SDF (Advantage Arrest, Elevate Oral Care, FL, USA) applied to the lesion at baseline and after 6 months [31] or NSF applied to the lesion once at baseline [19, 24]. In both groups, there was no removal of caries or unsupported enamel. Teeth were cleaned and isolated using cotton rolls. Dry cotton pellets were used to dry the lesions.

In the SDF group, the lips and perioral area were covered with petroleum jelly to protect against staining. In a plastic dappen dish, only one drop of SDF was dispensed. A single use microbrush was soaked in SDF and dabbed to remove excess before painting the affected tooth for 10 s. Cotton pellets were used to eliminate the excess. The solution was left to dry for one minute before allowing the child to close their mouth [32].

#### NSF group

##### Preparation

NSF was prepared in the labs of the Faculty of Pharmacy, Alexandria University following the Targino et al. method [20]. AgNPs were synthesized in an aqueous solution by the chemical reduction of silver nitrate (AgNO_3_) with sodium borohydride (NaBH4) and chitosan biopolymer as a stabilizing agent. Chitosan (28.7 ml, 2.5 mg/ml) was dissolved in 1% acetic acid overnight then AgNO_3_ (1 ml, 0.11 mol/L) was mixed with a magnetic stirrer until homogeneous. The mixture was transferred to an ice-cold bath, and NaBH4 (0.3 mL, 0.8 M) was added slowly while aggressively shaking it. The solution changed from clear to pale yellow then red, indicating the reduction of Ag+. Finally, the flask was removed from the ice bath and sodium fluoride (NaF) (10,147 ppm of fluorine) was added to improve stability and cariostatic efficacy of the solution. Stirring was maintained overnight. NSF was stored at 4 °C in a dark PET bottle until further usage with shelf life reaching 3 years [19].

### Characterization

**Fig. 1:**
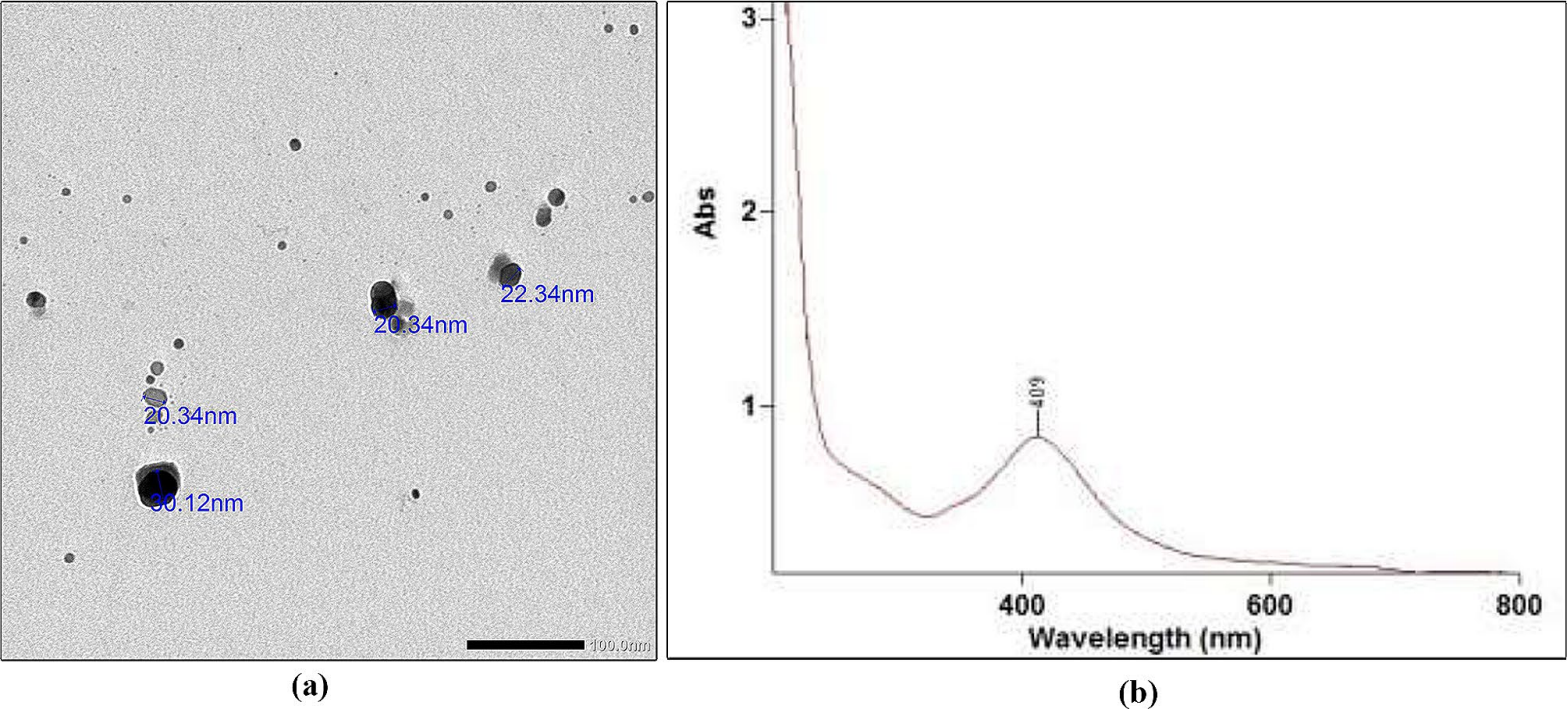
**(a)** Transmission electron microscope demonstrating shape and size of AgNPs in the prepared NSF. **(b)** UV- Vis spectrum of synthesized AgNPs

To determine the size and shape of AgNPs, field emission transmission electron microscope was used (JEOLJEM-2100 F) [33]. The electron micrographs revealed that most particles had spherical shapes ranging from 20 to 30 nm (Fig. 1a). AgNPs were also checked using UV/Visible spectrophotometer (Thermo Electron- Evolution 300) [24, 34] (Fig. 1b). The sample exhibited a peak with a particular absorbance at 409 nm wavelength, indicating that the average size of the existing AgNPs was 20 nm [35].

### Application

Two drops (10 mg) of NSF were applied on each tooth using a micro brush [19]. The drops were left in contact with the tooth surface for 2 min. The cavity was closed with a cotton pellet if possible by placing the pellet on the cavity, so that it would absorb the excess solution and allow the NSF to remain in contact with the lesion for longer time after application [7]. In both groups, the children were asked not to eat or drink for 30 min after applying the fluoride agents.

#### Outcome assessment

The primary outcome measure was caries arrest after 6 and 12 months. Lesion activity was assessed by the ICDAS criteria using visual and tactile inspection for surface discontinuities and texture. Before the study, three examiners received training for the calibration of caries examination using the online resources for ICDAS e-learning program by the ICDAS Foundation [36]. This was followed by clinical training in the dental clinic at the Department of Pediatric Dentistry and Dental Public Health, Faculty of Dentistry, Alexandria University, where 20 children were examined then re-examined after seven days to assess inter and intra-examiner agreement. The Cohen’s Kappa values for inter-examiner agreement among the three examiners were 0.86, 0.89 and 0.91 whereas the Kappa values for intra- examiner agreement were 0.90, 0.93 and 0.94 showing excellent agreement among examiners and across time [37].

Children were examined under natural daylight using a 0.5 mm ball-ended (CPI) probe and a disposable dental mirror. The teeth were cleaned and dried by a piece of dry gauze before the examination without magnification or radiographic assessment. The participants were assessed at baseline and after 6 and 12 months using the World Health Organization (WHO) criteria for caries experience (dmfs) [38] and ICDAS system for lesion severity and activity [39].

Lesions, that were moderate (ICDAS code 3 or 4) without visible dentine at baseline, were diagnosed as arrested after 6 or 12 months if they did not progress to a higher score (ICDAS code 5 or 6) [40]. Lesions, that were advanced (ICDAS code 5 or 6) with cavitated dentine at baseline, were diagnosed as arrested after 6 or 12 months if they were hard, smooth and not easily penetrated using light force on the probe [41–43]. The follow up examination was conducted by another calibrated assessor who was one of the three examiners.

The secondary outcome measure was parental satisfaction with the children’s dental appearance assessed by a self-rated questionnaire with responses on a 4-point Likert scale ranging from very satisfied, satisfied (combined for analysis into satisfied), unsatisfied, to very unsatisfied (combined for analysis into unsatisfied) [36]. Parents were also asked about adverse effects including tooth or gum pain, gum bleaching, gum swelling, and systemic toxicity such as nausea, vomiting, or generalized discomfort [13]. The staining of lesions was clinically assessed and recorded.

#### Sample size calculation

Sample size was estimated based on the primary clinical outcome, caries arrest, assuming 5% alpha error and 80% study power. Caries arrest rate after one application of NSF was previously reported to be 65.2% [24] whereas the arrest rate after biannual application of 38% SDF solution was calculated to be 49.6% based on the average of rates reported in previous studies [36, 41, 44]. The required number of participants per group was calculated by G power 3.0.10 to be 150. Considering a drop-out rate of 20%, the number of children to be recruited should be 180 in each group. Assuming an intra class correlation coefficient (ICC) = 0.3 [45] and an average number of carious lesions per child = 9 based on a pilot study conducted among a similar population, the design effect would be 3.4. The required number per group to accommodate the clustering of lesions within a child’s oral cavity = sample size* design effect [38] = 180*3.4 = 612 lesions per group. The number of lesions to be recruited should be at least 612 ≈ 620 in each group with a total of 1,240 lesions overall.

### Randomization, allocation concealment, and masking

Eligible children were randomly allocated to the study groups using a computer-generated random sequence, in a 1:1 ratio in blocks of 5 [46]. The allocation sequence was kept in opaque sealed envelopes by a trial-independent individual who was not participating in the study. Then each envelope was opened after completing the baseline clinical examination, ensuring eligibility and obtaining parental consent for the child’s participation in the study. The group the child was assigned to was identified as shown in the envelope and the child received the intervention. Masking was not possible because of the black staining caused by SDF.

### Statistical analysis

Data were analyzed using SPSS Statistics for Windows, Version 23.0 (SPSS Inc., Chicago, USA). The level of statistical significance was set at *p* < 0.05. Intention-to-treat analysis was used and analysis was conducted at lesion level. Carious lesions in children lost to follow up and surfaces that got restored or extracted were recorded as active lesions [36]. The primary outcome was also aggregated at the patient level for participants with multiple lesions. For a child with multiple carious lesions included in the study, a failure of any lesion at follow-up was considered patient-level arrest failure regardless of the status of other lesions. Arrest at surface and patient levels were compared between groups using Chi square test at 6 and 12 months.

Potential confounders as reported by caregivers were assessed by the Arabic version of the WHO questionnaire-child form [47, 48]. The questionnaire assessed the children’s demographic characteristics (age, sex, mother’s education) and oral health practices such as toothbrushing frequency, dental visits in the last year and sugar consumption at least once daily including eight types of sugary products: candies, biscuits and cakes, fruit, sugar-added chewing gums, jam and honey, carbonated beverages, sugar-sweetened milk and sweetened hot drinks. A sugar score was produced by summing the points of all sugary products that were consumed daily. The score ranged from 0 to 8 with higher scores indicating greater daily sugar consumption [49]. Oral hygiene was clinically assessed using the plaque index (PlI) of Silness and Loe [50] on 6 index teeth (#52, 55, 64, 72, 75, and 84) and averaging the scores to obtain the child’s score. Clinical and questionnaire data were collected using KoboTool Box, an open source data collection tool that allowed data collection in challenging conditions where the internet may not always be accessible [51].

The normal distribution of quantitative variables was assessed and Mann–Whitney U test/ independent samples t-test were used for between group comparisons as indicated. The chi-squared test was used to compare baseline demographic characteristics, oral hygiene practices, and caries arrest rate.

Multi-level multiple binary logistic regression was used to assess the effect of the intervention on caries arrest (arrested versus active) at surface level after 6 and 12 months controlling for confounders. Multi-level analysis accommodated the clustering of teeth within children since all eligible teeth per child were included. Intervention and confounders were introduced as fixed effect variables at two levels. Level 1 factors included lesion-related variables which were the type of intervention, tooth surface type (proximal, buccal/ lingual or occlusal) and baseline ICDAS code (classified into moderate lesions- ICDAS 3/ 4 and advanced lesions- ICDAS 5/ 6). Level 2 factors were child-related factors including child’s age, child’s sex, mother’s education, dental visits frequency, plaque index and sugar consumption score. The dmfs score was not included in the model due to collinearity with ICDAS codes. Toothbrushing frequency was also removed due to collinearity with the plaque index. Children were included as random effect variable. In addition, binary logistic regression was used to assess the effect of the intervention on ECC arrest at 6 and 12 months at the patient level controlling for all other confounders. Adjusted odds ratios (AORs), 95% confidence intervals (Cis) and p values were calculated.

## Results

**Fig. 2:**
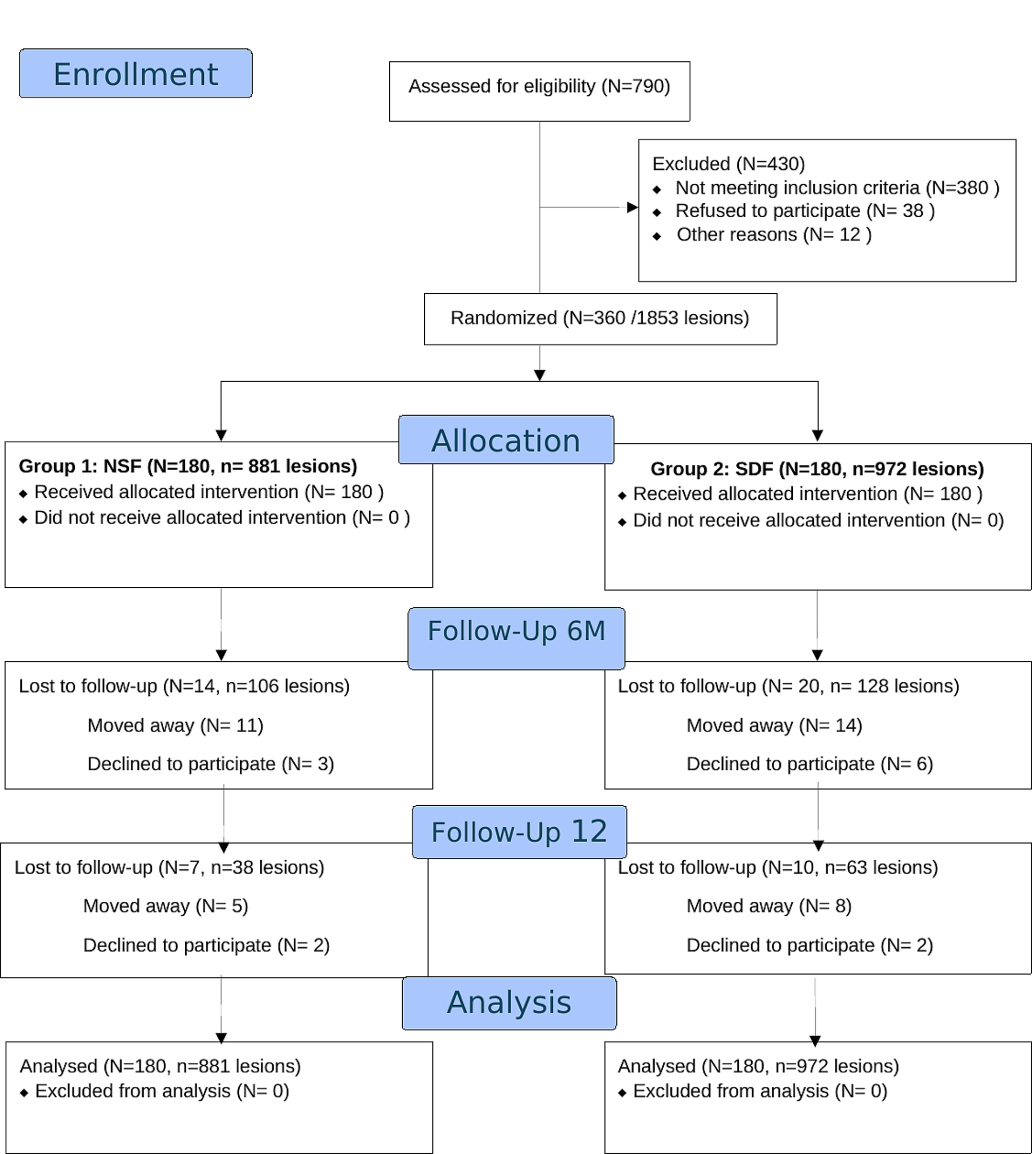
Flow chart of participants in the study

The study included 1853 active lesions in 360 children. One group had 180 children with 881 lesions treated with NSF and another group had 180 children with 972 lesions treated with 38% SDF (Fig. 2). After 6 months, 34 participants were lost to follow-up: 14 (7.8%) in the NSF group and 20 (11.1%) in the SDF group. After 12 months, 17 participants were lost to follow-up: 7 (3.9%) in the NSF group and 10 (5.6%) in the SDF group. The main reasons for dropout were changing nurseries and families moving to other areas. Two children in the NSF group accidentally received SDF and were analyzed with the NSF group following the intention to treat approach.

Table 1 shows that the mean (SD) age of the children was 42.3 (8.2) months and 53.1% were males with no significant differences between groups in sex and age (*p* = 0.13 and 0.17). A significantly higher percentage of mothers in the NSF group had high school and more education than in the SDF group (77.8% and 55%, *p* < 0.001). There were no significant differences between groups in sugar consumption (*p* = 0.11), toothbrushing frequency (*p* = 0.07) or dental visits last year (*p* = 0.24). Children in the NSF group had significantly lower dmfs score than children in the SDF group (median = 5 and 11, *p* = 0.01) with no significant difference between groups in the plaque score (*p* = 0.12). There were no significant differences between groups in baseline lesion severity (*p* = 0.20), or tooth surface type (*p* = 0.96).

**Table 1:** Socio-demographic background, oral health practices, and clinical characteristics of participants at baseline

		NSF group	SDF group	*P* value
Child level factors				
Demographic background				
Age in months	Mean (SD)	43.4 (5.5)	42.2 (8.2)	0.13
Sex: N (%)	Female	78 (43.3%)	91 (50.6%)	0.17
	Male	102 (56.7%)	89 (49.4%)	
Mother’s Educational level: N (%)	Less than high school	40 (22.2%)	81 (45%)	< 0.001
	High school and more	140 (77.8%)	99 (55%)	
**Oral health-related habits**				
Sugar consumption score	Mean (SD)	6.45 (1.24)	6.66 (1.19)	0.11
Tooth brushing frequency: N (%)	< once per day	88 (48.9%)	105 (58.3%)	0.07
	Once or more per day	92 (51.1%)	75 (41.7%)	
Dental visits last year: N (%)	< once or never	82 (45.6%)	71 (39.4%)	0.24
	At least once	98 (54.4%)	109 (60.6%)	
**Clinical characteristics**				
Dmfs	Mean ± SD	9.98 ± 11.24	12.81 ± 9.94	0.01
	Median (IQR)	5 (12)	11 (13)	
Pl index	Mean ± SD	1.42 ± 0.56	1.51 ± 0.56	0.12
**Tooth level factors**				
Baseline lesion severity: n (%)	ICDAS 3 & 4	266 (30.2)	267 (27.5)	0.20
	ICDAS 5 & 6	615 (69.8)	705 (72.5)	
Tooth surface type: n (%)	Buccal/ lingual	281 (31.9)	314 (32.3)	0.96
	Proximal	369 (41.9)	401 (41.3)	
	Occlusal	231 (26.2)	257 (26.4)	

N: number of children, n: number of tooth surfaces

Table 2 shows that there was a significant difference between groups in the arrest rate at 6 and 12 months (*p* < 0.001 for both). The arrest rate was higher in the NSF than the SDF group at 6 months (78.4% and 65.0%) and at 12 months (71.3% and 56.3%).

**Table 2:** Caries arrest by NSF and SDF after 6 and 12 months at tooth surface level

		NSF group (*n*/*N*)	SDF group (*n*/*N*)	*P* value
**6 months**	**Arrested**	78.4% (691/881)	65.0% (632/972)	< 0.001*
	**Active**	21.6% (190/ 881)	35.0% (340/972)	
**12 months**	**Arrested**	71.3% (628/881)	56.3% (547/972)	< 0.001*
	**Active**	28.7% (253/881)	43.7% (425/972)	

n: number of arrested lesions

N: number of all included lesions

Table 3 shows that there was a significant difference in the arrest rate between groups at the patient level at 6 and 12 months (*p* < 0.001 for both). The arrest rate was higher in the NSF than the SDF group at 6 months (61.1% and 40.0%) and 12 months (58.3% and 35.6%).

**Table 3:** Caries arrest by NSF and SDF after 6 and 12 months at patient level

		NSF group (*n*/*N*)	SDF group (*n*/*N*)	*P* value
**6 months**	**Arrested**	61.1% (110/180)	40.0% (72/180)	< 0.001*
	**Active**	38.9% (70/ 180)	60.0% (108/180)	
**12 months**	**Arrested**	58.3% (105/180)	35.6% (64/180)	< 0.001*
	**Active**	41.7% (75/180)	64.4% (116/180)	

n: number of patients with arrested or active lesions.

N: number of all patients.

Table 4 shows the results of the multi-level multiple binary logistic regression for the effect of the intervention on ECC arrest at surface level. There were significantly higher odds of caries arrest for lesions treated with NSF than SDF at 6 months (AOR = 2.57, 95% CI: 1.55, 4.26, *p* < 0.001) and at 12 months (AOR = 3.27, 95% CI: 1.89, 5.67, *p* < 0.001). Also, there were significantly higher arrest rates at the buccal/ lingual (AOR = 3.38 at 6 months and AOR = 3.18 at 12 months) and proximal surfaces (AOR = 2.09 at 6 months and AOR = 1.97 at 12 months) than the occlusal surfaces (*p* < 0.001 for both time points). Lesions which were moderate at baseline had significantly higher chances of arrest than advanced lesions at 6 months (AOR = 1.46, *p* = 0.03) but not at 12 months (AOR = 1.07, *p* = 0.70).

**Table 4:** Multi-level multiple binary logistic regression for NSF effect compared to SDF on the arrest of ECC at surface level at 6 and 12 months

Level	Variables		6 months		12 months	
			AOR (95% CI)	*P* value	AOR (95% CI)	*P* value
**Child level factors**	Age		1 (0.96, 1.03)	0.80	0.96 (0.93, 1.00)	0.06
	Sex	Female	0.70 (0.44, 1.12)	0.13	0.67 (0.4, 1.10)	0.12
		Male	1		1	
	Mother education	< high school	1.10 (0.67, 1.84)	0.70	1.00 (0.58, 1.74)	0.98
		>= high school	1		1	
	Dental visiting	Yes	0.78 (0.48, 1.27)	0.32	0.97 (0.58, 1.63)	0.91
		No	1		1	
	Sugar score		0.93 (0.77, 1.14)	0.49	1 (0.81, 1.23)	0.97
	Plaque index		0.99 (0.65, 1.51)	0.97	0.71 (0.45, 1.12)	0.14
**Lesion level factors**	Intervention	NSF	2.57 (1.55, 4.26)	< 0.001	3.27 (1.89, 5.67)	< 0.001
		38% SDF	1		1	
	Lesion surface	Buccal/ lingual	3.38 (2.28, 5.01)	< 0.001	3.18 (2.15, 4.72)	< 0.001
		Proximal	2.09 (1.47, 2.98)		1.97 (1.37, 2.83)	
		Occlusal	1		1	
	Baseline severity (ICDAS code)	Moderate (ICDAS 3/4)	1.46 (1.03, 2.07)	0.03	1.07 (0.76, 1.51)	0.70
		Advanced (ICDAS 5/6)	1		1	

AOR: adjusted odds ratio, CI: confidence interval

Table 5 shows that there were significantly higher odds of caries arrest in patients treated with NSF than SDF (at 6 months, AOR = 1.71, 95% CI: 1.10, 2.65, *p* = 0.017, at 12 months, AOR = 2.37, 95% CI: 1.52, 3.69, *p* < 0.001). All other variables, including demographic factors, oral health habits, or plaque index score were not significantly associated with arrest at 6 or 12 months.

**Table 5:** Binary logistic regression for NSF effect compared to SDF on the arrest of ECC at patient level at 6 and 12 months

Variables		6 months		12 months	
		AOR (95% CI)	*P* value	AOR (95% CI)	*P* value
**Age**		1.02 (0.99, 1.05)	0.22	1 (0.96, 1.03)	0.71
**Sex**	**Female**	0.96 (0.63, 1.48)	0.87	0.73 (0.47,1.12)	0.15
	**Male**	1		1	
**Mother education**	**< high school**	0.72 (0.45, 1.14)	0.16	0.87 (0.54,1.41)	0.58
	**>=high school**	1		1	
**Dental visiting**	**Yes**	0.74 (0.48, 1.16)	0.19	1 (0.64,1.54)	0.97
	**No**	1		1	
**Sugar score**		0.90 (0.76, 1.08)	0.27	0.96 (0.80,1.15)	0.66
**Plaque index**		0.78 (0.53, 1.16)	0.22	0.74 (0.5 ,1.10)	0.13
**Intervention**	**NSF**	1.71 (1.10, 2.65)	0.017	2.37 (1.52, 3.69)	< 0.001
	**38% SDF**	1		1	

AOR: adjusted odds ratio, CI: confidence interval

**Fig. 3:**
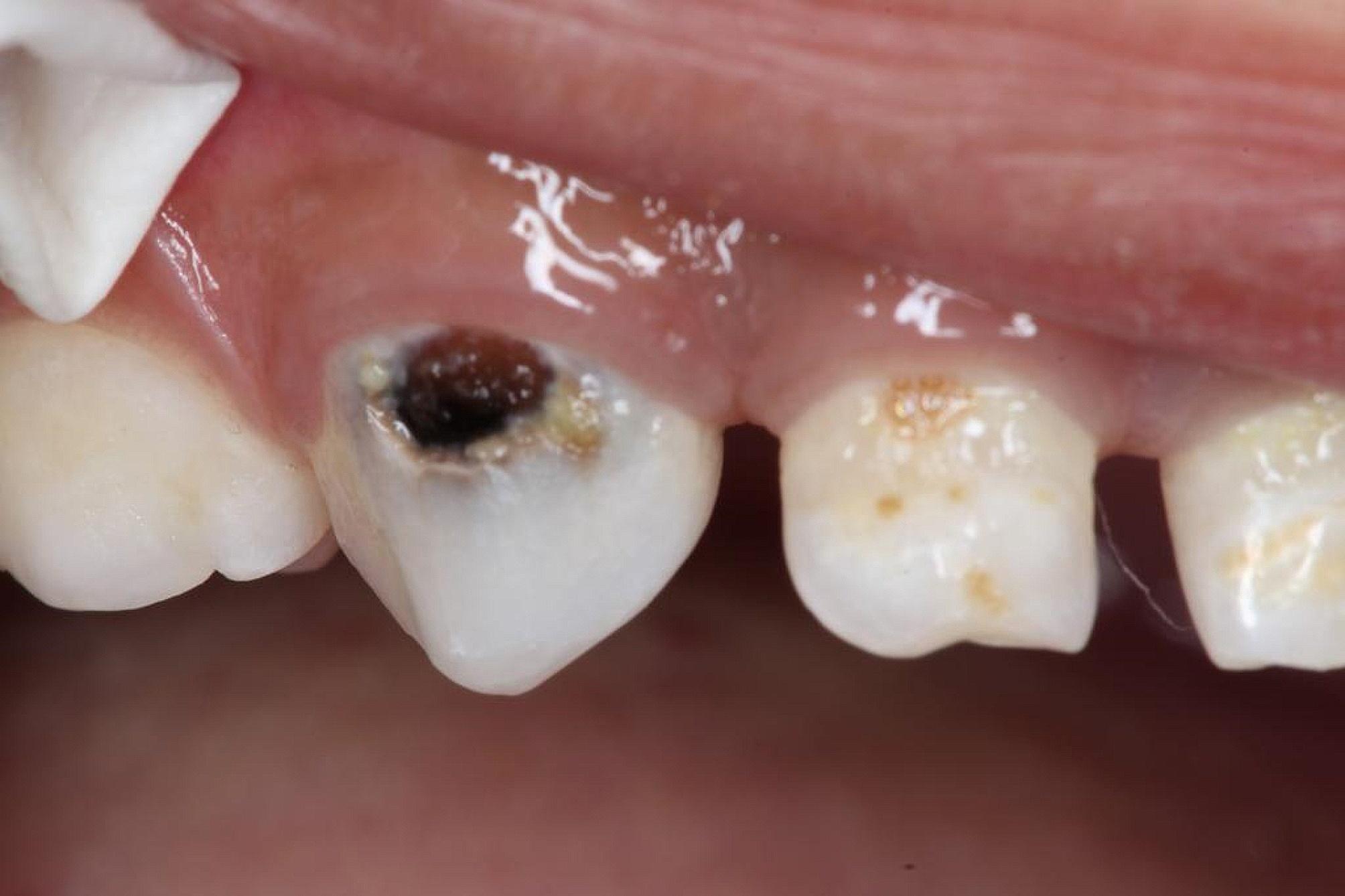
Arrested caries after SDF application

**Fig. 4:**
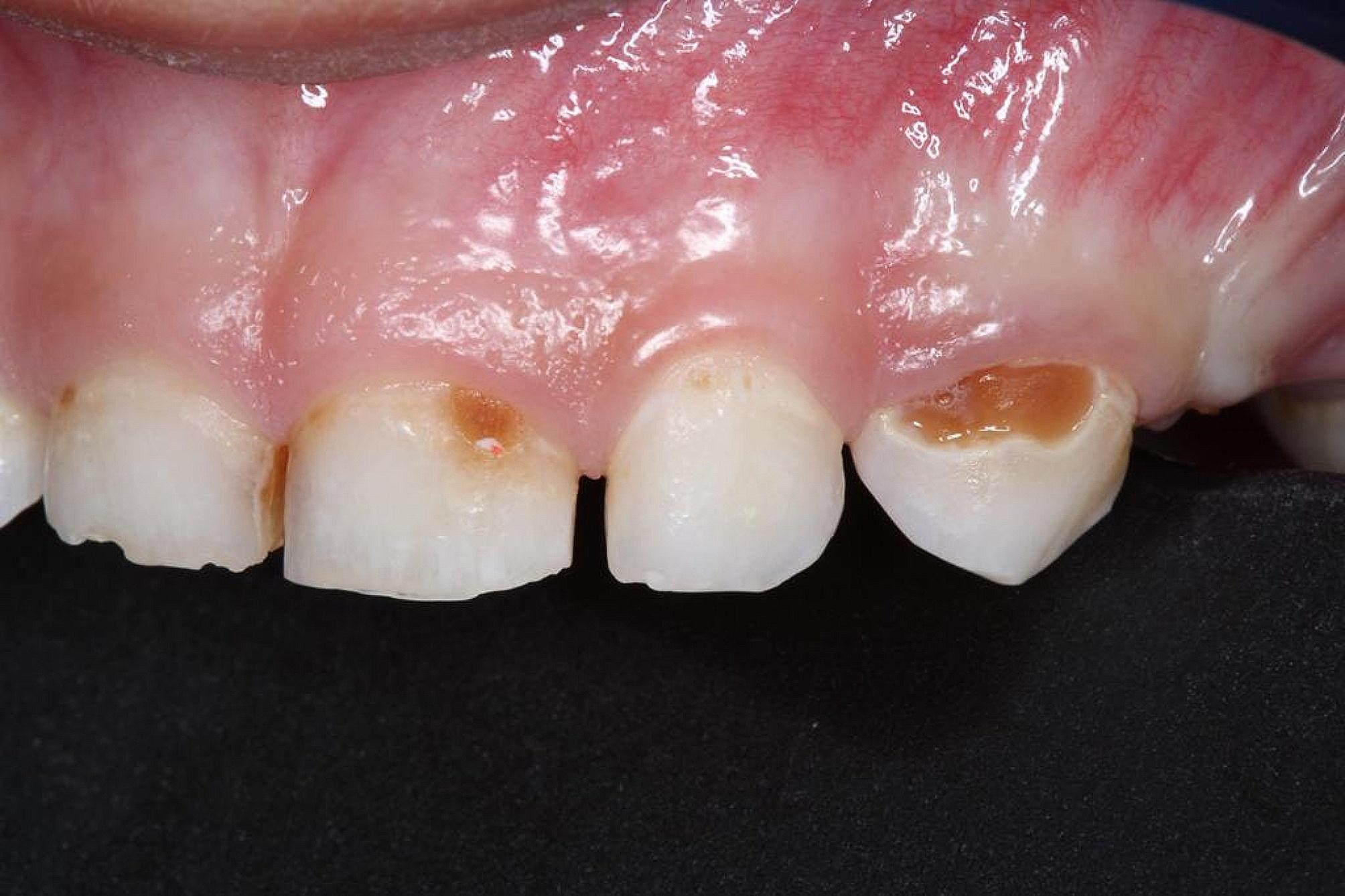
Arrested caries after NSF application

All carious lesions in the SDF group were stained black (Fig. 3) compared to none in the NSF group (Fig. 4). Also, 2.8% of parents reported tooth and gum pain and 13.3% reported gum bleaching in the SDF group only. There were no reports of gum swelling, acute systemic illness such as nausea or vomiting or major adverse effects in both groups. A significantly greater percentage of parents in the NSF than the SDF group were satisfied with children’s dental appearance: 97.2% and 76.1%, *p* < 0.001.

## Discussion

The study showed that, after 6 and 12 months, the ECC arrest rate of NSF was significantly higher than 38% SDF in children who were younger than 4 years, with high caries severity and living in rural areas around Alexandria, Egypt at both surface and patient level. Thus, the null hypothesis of the study was rejected. Also, there was greater parental satisfaction with the children’s dental appearance in the NSF than the SDF group. No adverse events or systemic manifestations were observed in NSF group whereas gum pain and bleaching were reported in few children in the SDF group.

The study had some limitations. The outcome assessor could not be masked because of the black discoloration caused by SDF. In addition, because of the rural setting and similar to previous studies [51, 53, 54], we did not use radiographic assessment of caries lesions and, therefore, some lesions may have been misclassified. On the other side, the study had many strengths. The outcome was assessed using the ICDAS criteria which is based on lesion stability as opposed to assessing arrest based on hardness and color, which is more subjective. Compared to histological classification, the ICDAS system allowed for accurate estimation of coronal caries activity [55, 56]. The rural setting supports the generalizability of findings to individuals most in need of minimally invasive dental care. Further studies with longer follow-up periods are required to assess the effectiveness of NSF as preventive and caries arresting agent.

The findings showed an arrest rate of NSF after 6 months similar to that reported by Tirupathi et al. (80%) [7] although it was slightly higher than that described by dos Santos et al. (72.7%) and Nagireddy et al. (73%) [19, 24]. The higher arrest rate in our study may be because other studies included caries only at the dentine level while the present study included enamel and dentine cavitated lesions. Furthermore, in this study the arrest rate of NSF after 12 months (71.3%) was relatively in line with the results of these studies (7,19,24).

The study showed that the arrest rate of NSF was significantly higher than SDF at tooth surface level and patient level, at 6 and 12 months. There is scarce literature comparing the effectiveness of NSF and SDF clinically. One clinical trial [7] showed that there was a slightly better cariostatic effect after NSF than SDF in primary carious molars after 6 and 12 months, although the difference was not significant. The difference between the two studies may be because of the different preparation technique for NSF and caries assessment where the other study [7] used the Mount and Hume classification of caries. In addition, the other study [7] included only primary molars, instead of all teeth in the present study. Despite these differences, the authors reported an arrest rate of NSF similar to our study. In contrast, Al-Nerabieah et al. [52] reported that NSF had lower arrest rate than SDF in dentin lesions of primary teeth although the difference was not significant. They used a different protocol to apply the agent, applying a single drop of NSF on the lesion surface, instead of two drops following the standard NSF application protocol [19, 24, 57]. In addition, Al-Nerabieah et al. [52] used the Nyvad criteria to assess caries instead of ICDAS.

The present results showed that the arrest rate of NSF was decreased at 12 months compared to 6 months, and this finding was consistent with those reported by others [7, 19, 24]. This may be attributed to the application protocol of NSF which was done only once in 12 months.

The effectiveness of NSF in ECC arrest can be attributed to the small size of the AgNPs and their spherical shapes which increase the contact surface [35, 46, 58]. The average AgNPs size in this study was 20 nm, as opposed to sizes less than 10 nm which raise concerns about biocompatibility [48, 59]. Thus, the size we used ensured the biocompatible and antimicrobial effects and posed no threat to safety [35, 48, 60]. This may explain why no adverse effects were reported in the NSF group and agrees with trials following-up NSF for up to one year and reporting no side effects [7, 19, 20, 24, 61].

The study showed that NSF had greater effectiveness than SDF, without the disadvantages of SDF like black staining that affects parental satisfaction. Besides, NSF has no metallic taste like SDF [51]. Thus, NSF can be recommended for ECC arrest especially in anterior teeth where esthetics may be of greater concern. NSF is a viable option for the minimally invasive management of ECC in settings with high caries severity and sugar exposure like the present study. Further studies are needed to compare the cost-effectiveness of both agents to make informed recommendations.

## Conclusion

NSF demonstrated greater effectiveness in arresting ECC in children with no black staining of teeth, metallic taste, or ulceration, and with greater parental satisfaction than SDF. NSF can be recommended to arrest ECC lesions in children especially in underprivileged communities with further investigation needed to assess its cost effectiveness.

## Data Availability

All data included in this study are available from the corresponding author upon reasonable request.

## Abbreviations

SDF: Silver Diamine Fluoride
NSF: Nano silver fluoride
ECC: Early childhood caries
ICDAS: International Caries Detection and Assessment System
AgNPs: Silver nanoparticles
NaF: Sodium fluoride
CPI: Community periodontal index
PI: Plaque index
SPSS: Statistical Package for Social Sciences
WHO: World Health Organization
